# Large language models and humans converge in judging public figures’ personalities

**DOI:** 10.1093/pnasnexus/pgae418

**Published:** 2024-09-19

**Authors:** Xubo Cao, Michal Kosinski

**Affiliations:** Graduate School of Business, Stanford University, Stanford, CA 94305, USA; Graduate School of Business, Stanford University, Stanford, CA 94305, USA

**Keywords:** personality perception, zero-shot predictions, large language models, AI

## Abstract

ChatGPT-4 and 600 human raters evaluated 226 public figures’ personalities using the Ten-Item Personality Inventory. The correlation between ChatGPT-4 and aggregate human ratings ranged from *r* = 0.76 to 0.87, outperforming the models specifically trained to make such predictions. Notably, the model was not provided with any training data or feedback on its performance. We discuss the potential explanations and practical implications of ChatGPT-4's ability to mimic human responses accurately.

Large language models (LLMs) are trained on huge text corpora to predict the next word in a sequence. Through this process, they not only learn grammatical rules and semantic associations (1) but also develop a wide array of other capabilities. These include translating between languages (2), solving reasoning and mathematical tasks (3), and distinguishing between the mental states of different characters in a story (4). Importantly, none of these capabilities were deliberately engineered or anticipated by their creators. Instead, they emerged as by-products of LLMs’ training to predict the next word in a sentence (5).

In this study, we explore yet another unexpected capability of LLMs: their ability to predict people's perceptions of public figures’ personalities. Given the extensive and diverse training data available to these models—encompassing social media posts, Wikipedia articles, press stories, and books—it is plausible that LLMs would develop such an ability. These data sources are rich in opinions about public figures and descriptions of their actions and statements. While these cues may not accurately reflect the actual personalities of public figures, they do not only reflect but also shape public perceptions. Furthermore, LLMs could greatly benefit from encoding such perceptions. For example, completing the sentence “Laura decided to vote for Trump despite perceiving him to be overly ______” requires recognizing that the 45th U.S. President is often perceived as disagreeable and contentious (6).

Previous research suggests that LLMs’ semantic spaces mirror public perceptions of figures’ personalities (7, 8). For instance, Cao and Kosinski (8) demonstrated that public figures’ perceived personalities could be inferred from their names’ locations in GPT-3's semantic space. Similar approaches have been employed to gauge perceived leadership skills (7). However, extracting such predictions traditionally required extensive training data and developing predictive models—a time-consuming and laborious process. For example, Cao and Kosinski's study (8) involved extracting embeddings for 300 public figures’ names from OpenAI's Application Programming Interface (API), recruiting 600 judges to assess these figures using the Ten-Item Personality Inventory (TIPI; (9)), aggregating their responses into perceived personality profiles, and then training a cross-validated ridge regression model to predict figures’ profiles from their names’ embeddings.

This article demonstrates that LLMs’ increasing ability to follow instructions eliminates the need for collecting training data and developing predictive models. Rather than relying on human judges and training predictive models, we employ a modern LLM, ChatGPT-4, to directly complete the TIPI, assessing public figures’ Big Five personality traits: openness, conscientiousness, extraversion, agreeableness, and emotional stability. This approach enables us to estimate the perceived personality of any public figure with sufficient representation in ChatGPT-4's training data. We still used the perceived personality profiles of 226 public figures collected in our previous research from human judges (8, 10), but only to validate the accuracy of ChatGPT-4's assessments—and not to train any models. (Data collection was approved by Stanford University IRB [protocol #59974].)

We adapted TIPI prompts and repeated them for each question (marked in *italics*) and for each public figure (indicated by underscored names):

“Here is a characteristic that may or may not apply to Donald Trump. Please indicate the extent to which most people would agree or disagree with the following statement: *I see Donald Trump as extraverted, enthusiastic.*

1 for Disagree strongly, 2 for Disagree moderately, 3 for Disagree a little, 4 for Neither agree nor disagree, 5 for Agree a little, 6 for Agree moderately, 7 for Agree strongly.

Answer with a single number.”

ChatGPT-4 was reset after each question to reduce consistency bias and independently evaluate each personality dimension. Default parameter settings were used (e.g. temperature = 1). ChatGPT-4's responses were aggregated to compute five trait scores for each public figure. This procedure was repeated 10 times to assess the internal consistency of ChatGPT-4's evaluations. The single-rater intra-class correlation coefficient across these 10 runs ranged from 0.95 to 0.98 for the five traits, indicating highly consistent predictions.

Figure 1 shows that ChatGPT-4's accuracy (blue bars) ranged from *r* = 0.76 for openness to *r* = 0.87 for agreeableness. These results are comparable to the accuracy of regression models specifically trained in our previous work (8) to approximate human ratings (orange bars). The accuracy is remarkable, considering that ChatGPT-4 was neither explicitly trained for these tasks nor provided with feedback on its performance. To put this in perspective, ChatGPT-4's ratings were better predictors of average human ratings than individual human ratings themselves, which correlated with aggregate ratings at *r* = [0.56 to 0.66]. Furthermore, ChatGPT-3.5 achieved much lower accuracy (*r* = [0.25 to 0.77]; see Supplementary material).

**Fig. 1.**
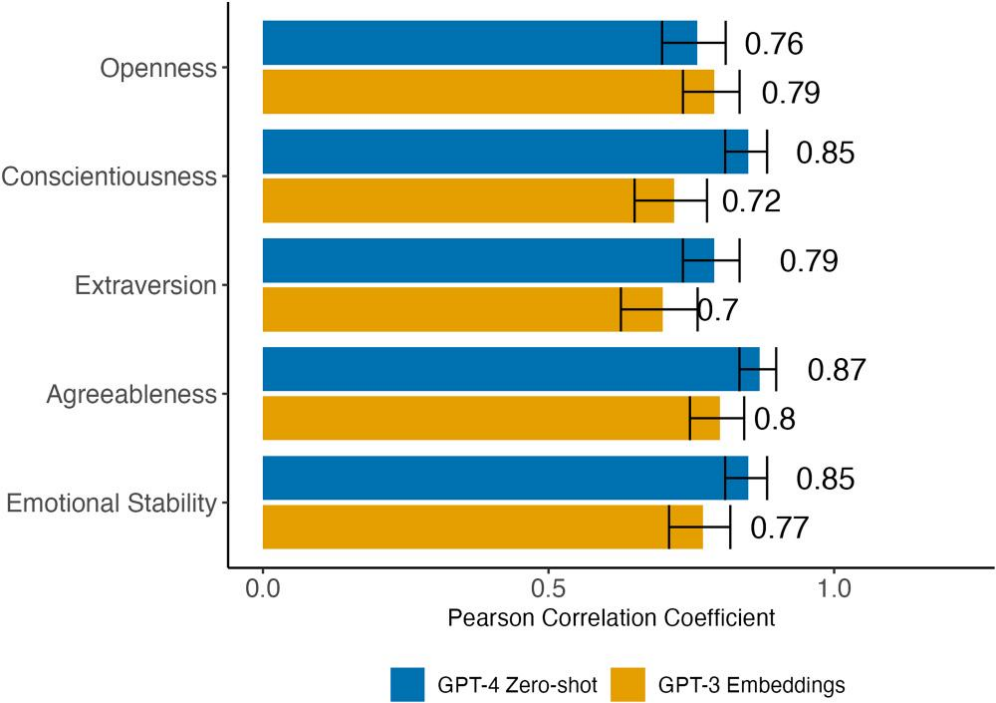
Accuracy of ChatGPT-4's (blue bars) TIPI ratings of public figures’ personalities. Accuracy was averaged across 10 runs, applying Fisher's Z-transformation. The accuracy of embedding-based regression models observed in previous research (8) is provided for context (orange bars). In both cases, aggregate human ratings served as the ground truth. Error bars represent 95% CIs. All correlations are significant at *P* < 0.001.

Figure 2 shows that the similarity between human and ChatGPT-4 judgments was higher for well-known individuals, likely because both humans and ChatGPT-4 encountered more relevant cues in their respective training data.

**Fig. 2.**
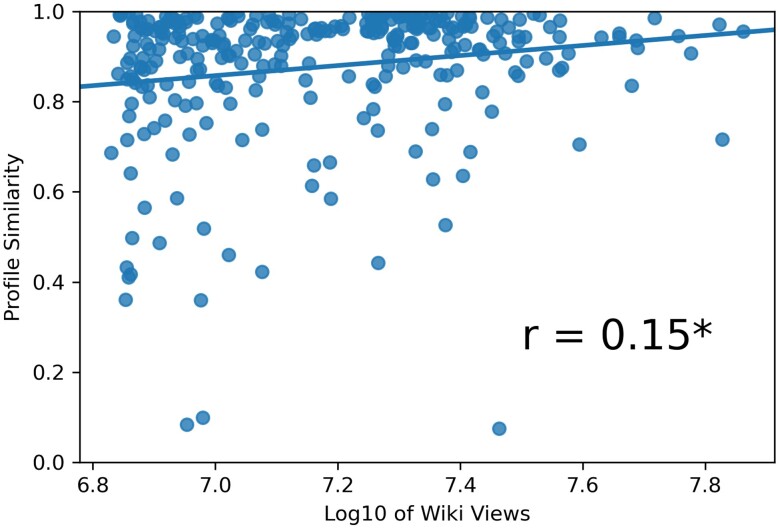
The profile similarity (Pearson correlation) between human and ChatGPT-4 ratings of each public figure as a function of Wikipedia page views (log-transformed), a proxy for public figures’ popularity. One outlier with extreme negative similarity was omitted. These two variables correlated at *r* = 0.15 (*P* < 0.05).

ChatGPT-4's ratings had high face validity, as illustrated by the top and bottom 10 public figures rated on each trait (Table 1). For example, artists and writers like Miley Cyrus and Stephen King were perceived as highly open-minded, liberal, creative, and artistic. At the other extreme were autocrats like Kim Jong-un and conservative politicians like Dick Cheney.

**Table 1.** Top and bottom 10 public figures according to their predicted perceived traits.

	Public figures	
	Bottom (ascending)	Top (descending)
Agreeableness	Joseph Kony, Jim Jones, Saddam Hussein, Charles Manson, Kim Jong-un, Osama bin Laden, Zodiac Killer, Kim Jong-il, Muammar al-Gaddafi, Heinrich Himmler	Emma Watson, Walt Disney, Mother Teresa, Rosa Parks, Pope John Paul II, Anne Frank, Audrey Hepburn, Julia Child, Jackie Robinson, Neil Patrick Harris
Conscientiousness	Ed Gein, Charles Manson, Charlie Sheen, Lindsay Lohan, Amy Winehouse, Lee Harvey Oswald, Anna Nicole Smith, Jeffrey Dahmer, Donald Trump, Joseph Kony	Yao Ming, Usain Bolt, The Rock, Beyoncé, Will Smith, Bruce Lee, Pope John Paul II, Bear Grylls, Grace Hopper, Kaká
Emotional Stability	Kurt Cobain, Jim Jones, Howard Hughes, Donald Trump, Mark David Chapman, Anna Nicole Smith, Lindsay Lohan, Joseph Kony, Mario Barwuah Balotelli, Jeffrey Dahmer	Pelé, Warren Buffett, Clint Eastwood, Neil Armstrong, Elizabeth II of the United Kingdom, Condoleezza Rice, Carl Sagan, Bob Marley, Mother Teresa, Pope John Paul II
Extraversion	Howard Hughes, Jeffrey Dahmer, Ed Gein, Zodiac Killer, Stephen Hawking, Elizabeth II of the United Kingdom, Pope Benedict XVI, Heinrich Himmler, Kristen Stewart, Mark David Chapman	Hulk Hogan, Julia Child, Jim Carrey, Shaquille O'Neal, Lady Gaga, Lil Wayne, Bear Grylls, Kesha, Vince McMahon, Nicki Minaj
Openness	Kim Jong-un, Joseph Kony, Josef Mengele, Saddam Hussein, Dick Cheney, Heinrich Himmler, Jim Jones, Bernard L. Madoff, Sarah Palin, Rick Santorum	Miley Cyrus, Heath Ledger, Joss Whedon, Jon Stewart, Les Paul, Ric Flair, Stephen King, Jennifer Lopez, Roger Federer, Justin Timberlake

Complete lists at: https://osf.io/3nfu6/.

The similarity between LLM and human responses raises intriguing questions about the underlying processes. It is well established that humans not only tend to agree with each other when judging others’ personalities, but their judgments also often reflect the actual personalities of the targets (11). This high consistency and validity of human judgment rely on a complex interplay of psychological mechanisms. Humans must identify and interpret relevant personality cues, store the resulting judgments, and retrieve them when needed. They also need to be able to follow instructions when responding to questionnaires designed to capture these impressions. The high degree of similarity between ChatGPT-4's responses and human judgments suggests that the model has developed an ability to replicate the effects of these psychological processes.

One possible explanation is that LLMs reproduce these effects by superficially mimicking the outcomes of human psychological processes. The training data for LLMs are replete with human depictions of public figures’ personalities, which are shaped by psychological processes such as social cognition, theory of mind, lay personality theories, impression formation, and stereotypes. LLMs might learn to imitate these human perceptions by reproducing patterns observed in the data—such as the frequent pairing of “Trump” with “extraverted”—without actually engaging in any analogous psychological processes. This idea aligns with John Searle's famous Chinese Room argument, which suggests that a system could behave as if it understands Chinese (or, in this case, as if it had personality perceptions) without possessing any human-like mental capacities (12).

However, the resemblance between LLM and human responses might not be purely superficial. It is conceivable that as LLMs are trained to emulate human language, they replicate some underlying psychological mechanisms. For instance, the semantic spaces that LLMs use to encode information about word meanings and relationships have been found to resemble human semantic memory, grouping words by meaning rather than by superficial features like co-occurrence, spelling, or phonetics (1). Similarly, research has shown that public figures’ names tend to co-occur with adjectives that reflect their perceived personality traits (7). For example, figures perceived as disagreeable—such as Donald Trump, Kim Jong-il, and Vladimir Putin—are often closely associated with terms like “insulting,” “intimidating,” “abusive,” and “controversial.” This suggests that LLMs’ personality perceptions might be enabled by emergent processes that share some similarities with the psychological mechanisms underlying human perceptions (7, 8).

Regardless of the underlying mechanisms, ChatGPT-4's performance is remarkable. While previous research has shown that specialized models can achieve similar outcomes, their development is costly and time-consuming. In contrast, ChatGPT-4 produced comparable results at minimal cost and without any specific training. The accuracy could be further improved by aggregating multiple judgments for each public figure. Moreover, adding judgments of other LLMs could mitigate biases inherent in their respective training datasets. While our study focuses on public figures, this method could also be applied to individuals absent from the training data, for example, by analyzing samples of text written by or about the individual in question.

The same method could predict perceptions of other traits, such as likability, perceived intelligence, or political orientation. Accurate, reliable, and accessible predictions of public perceptions could revolutionize fields that rely on such insights, such as management and political science. Moreover, the similarity between the model and human responses bolsters the growing evidence suggesting that LLMs can serve as a viable proxy for human participants in social science research (13).

## Supplementary Material

pgae418_Supplementary_Data

## Data Availability

Our data and code are available at: https://osf.io/3nfu6/.

## Preprints

This manuscript was posted as a preprint at: https://psyarxiv.com/zbhyk/.
