# Supplementary material for: Large language models and humans converge in judging public figures’ personalities
Source: PNAS Nexus. 2024 Sep 19;3(10):pgae418. doi: 10.1093/pnasnexus/pgae418 (PMC11443023; doi:10.1093/pnasnexus/pgae418)
Supplement: pgae418_Supplementary_Data [file pgae418_supplementary_data.docx]

# *Large Language Models and Humans Converge in Judging Public Figures' Personalities*

Xubo Cao, Michal Kosinski (michalk@stanford.edu)

**Human Ratings**. The predictive validity of ChatGPT-4’s responses was examined using human ratings collected in previous research (8). We started with the 300 (out of 11,341) most popular public figures from 43 countries listed in the Pantheon 1.0 dataset (10). Their popularity was approximated by their Wikipedia page views. As artists were particularly popular, we limited their number to 100 to include public figures from other domains. Figures born before 1900 were excluded, as the raters may have been less familiar with them. 600 raters recruited on Prolific.com completed TIPI (9) for ten randomly chosen public figures (they could skip unfamiliar figures). Public figures received ratings from 18.89 raters (SD=10.38) on average. 74 public figures rated by fewer than ten raters were removed to maintain the reliability of our measure.

**ChatGPT-3.5 Performance.** The same approach was used to test ChatGPT-3.5’s performance (“gpt-3.5-turbo-1106”). Its responses were less consistent, with the single-rater intraclass correlation coefficient (ICC) spanning from 0.51 to 0.88 (ICC for ChatGPT-4 was from 0.95 to 0.98). As shown in Figure S1, ChatGPT-3.5 was less accurate than ChatGPT-4 across all traits.

**
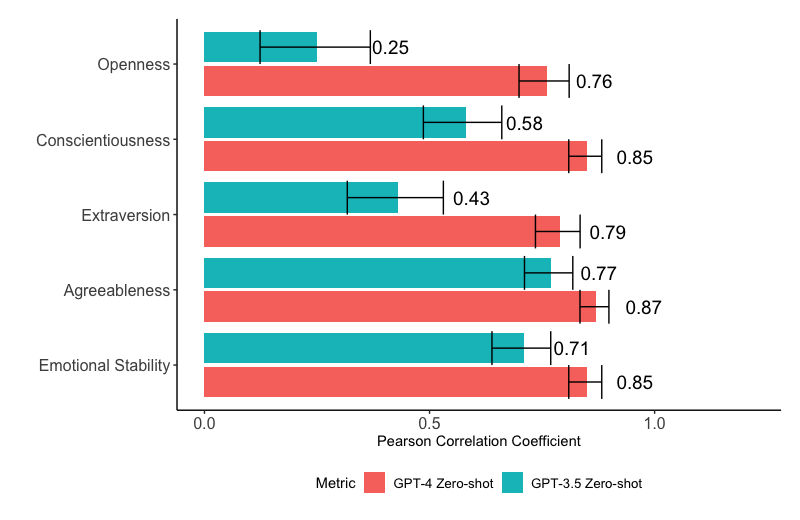
**

**Figure S1.** ChatGPT-3.5 and ChatGPT-4 accuracy at predicting public figures’ perceived personalities. Error bars represent 95% confidence intervals. All correlations are significant at the p<.001 level.
